# Bioluminescent Systems for Theranostic Applications

**DOI:** 10.3390/ijms25147563

**Published:** 2024-07-10

**Authors:** Hyemin Kim, Seung Oh Jung, Seungchan Lee, Yujin Lee

**Affiliations:** Department of Cosmetics Engineering, Konkuk University, 120 Neungdong-ro, Gwangjin-gu, Seoul 05029, Republic of Korea; autre4@konkuk.ac.kr (S.O.J.); ranny98@konkuk.ac.kr (S.L.); yujani43@konkuk.ac.kr (Y.L.)

**Keywords:** bioluminescence, luciferase, theranostics, bioimaging, biosensing, photodynamic therapy, optogenetics

## Abstract

Bioluminescence, the light produced by biochemical reactions involving luciferases in living organisms, has been extensively investigated for various applications. It has attracted particular interest as an internal light source for theranostic applications due to its safe and efficient characteristics that overcome the limited penetration of conventional external light sources. Recent advancements in protein engineering technologies and protein delivery platforms have expanded the application of bioluminescence to a wide range of theranostic areas, including bioimaging, biosensing, photodynamic therapy, and optogenetics. This comprehensive review presents the fundamental concepts of bioluminescence and explores its recent applications across diverse fields. Moreover, it discusses future research directions based on the current status of bioluminescent systems for further expansion of their potential.

## 1. Introduction

Light-based diagnosis and therapy have achieved significant advancements in healthcare innovation due to their versatility and minimal invasiveness [1,2]. Based on light–tissue interactions, modern medical imaging systems can obtain several different kinds of information, including the composition, structure, and functional features of tissues, as well as tissue abnormalities and insights into disease progression [3,4]. Light can also serve as a therapeutic agent, generating heat or triggering photochemical and biological reactions. Various kinds of light-based therapies, such as photothermal therapy (PTT) [5], photodynamic therapy (PDT) [6], and photobiomodulation (PBM) [7], have been developed for diverse applications, including anticancer therapy [8,9] and tissue regeneration [10,11]. Owing to its patient compliance and therapeutic efficacy, light-based theranostics has emerged as a mainstream in recent healthcare.

To effectively exploit light-based theranostic systems, delivering light into deep tissues is considered a critical issue. Thus, light-based approaches have utilized near-infrared (NIR) light due to its relatively deep tissue penetration [12]. Various strategies have also been employed, including the implantation of miniaturized light sources such as micro light-emitting diodes (µLEDs) [13], as well as the integration of light-delivering materials like optic fibers [14] and waveguides [15]. Among these strategies, bioluminescence is regarded as a promising alternative light source for theranostic applications, offering minimally invasive light delivery. Bioluminescence, a kind of chemiluminescence, arises from the activity of the luciferase enzyme of living organisms [16,17]. Because light can be produced by biochemical reactions within tissue, it does not require invasive procedures and it is not constrained by penetration depth limitations. For example, the oxidation of D-luciferin by firefly luciferase yields light around 560 nm, and it has been widely used in medical imaging and bioluminescence assays [18,19,20].

**Figure 1:**
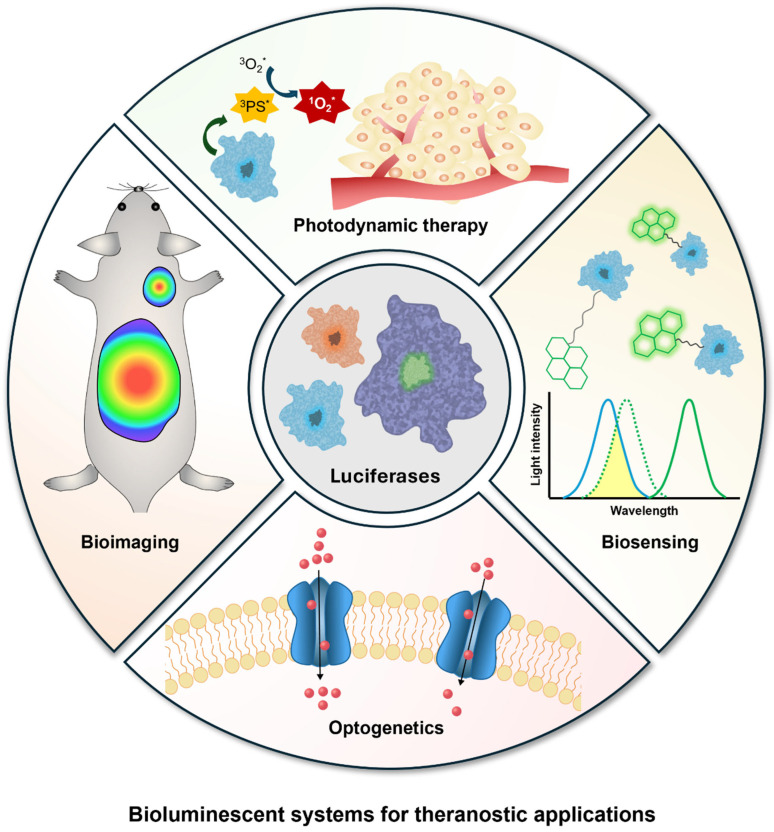
Overview of bioluminescent systems for theranostic applications. Unpaired electrons are represented by an asterisk (*).

As light-based theranostic approaches continue to evolve, numerous studies have explored the utilization of bioluminescence. This comprehensive review presents the diverse forms of bioluminescence-based systems and their theranostic applications. More than 40 bioluminescence pathways, involving specific luciferins and luciferases, have been reported, and a few of them are actively used in biomedical applications [21,22]. Luciferase can be introduced into target cells via genetic transduction using plasmid vectors to ensure stable expression, or it can be delivered as a recombinant protein with delivery systems designed to protect it from the surrounding environment. Bioluminescence itself can facilitate photochemical and biological reactions, but it can also induce the emission of longer-wavelength light through bioluminescence resonance energy transfer (BRET). These various kinds of bioluminescence-based systems have been exploited for diverse theranostic applications, and their recent advancements in bioimaging, biosensing, photodynamic therapy, and optogenetic applications are presented in this review (Figure 1). Finally, we propose future research directions for the improvement of the current bioluminescence-based system, outlining their potential contribution to future medicine.

## 2. Bioluminescence-Based Systems

### 2.1. Luciferases and Luciferins

As mentioned earlier, diverse luciferases are available for theranostic applications. While these enzymes share common features that they can produce light through a catalytic reaction, each luciferase utilizes a specific luciferin substrate within its optimal environmental conditions. In addition, the light emission kinetics and wavelength are unique for each enzyme. These characteristics are crucial factors to consider when selecting the most suitable bioluminescence system for a desired application. Therefore, this section provides a brief explanation of the characteristics of representative luciferins and luciferases used for theranostic applications.

The firefly luciferase (FLuc)–D-luciferin pair is one of the most widely used bioluminescence systems. FLuc catalyzes the oxidation of D-luciferin in the presence of ATP, Mg^2+^, and oxygen. This reaction activates D-luciferin through adenylation, leading to the formation of the excited state of oxyluciferin, which emits light around 560 nm as it relaxes to its ground state [23]. Over 40 firefly species utilize D-luciferin as a substrate, and the *Photinus pyralis* firefly is the most common source in research [24]. The D-luciferin bioluminescence system has several unique features that encourage its wide use. The requirement for ATP in the reaction might seem like a limitation, but it can be beneficial in applications. Because it is less susceptible to background signals from spontaneous oxidation, it can lead to clear results with less interference. The D-luciferin bioluminescence system also exhibits a remarkable quantum yield of around 40% with a long emission [25]. Finally, D-luciferin has good aqueous solubility and lower toxicity [21,26], which encourages its active use as a valuable tool in theranostic applications.

Coelenterazine is also a common substrate for luciferases, generally found in aquatic organisms. *Renilla* luciferase (RLuc), *Gaussia* luciferase (GLuc), and *Oplophorus* luciferase (OpLuc) catalyze the oxidation of coelenterazine to the excited state of coelenteramide in the presence of oxygen [27]. In the RLuc and GLuc reactions, the excited intermediate emits light around 480 nm as it relaxes to its ground state. Unlike the FLuc–D-luciferin pair, the bioluminescence reaction with coelenterazine does not rely on ATP, which can be beneficial for bioimaging in ATP-depleted environments. However, it is vulnerable to spontaneous oxidation, potentially causing undesired background signals [28]. Coelenterazine also has low aqueous solubility, making it less suitable for certain applications compared to D-luciferin. The kinetics of bioluminescence produced by coelenterazine is characterized by a flash rather than a glow, with the behavior dependent on specific luciferases. RLuc typically exhibits a relatively slow rise in light intensity followed by a long decay period, while GLuc shows a rapid rise in light intensity followed by a quick fall [29]. Bioluminescence produced by OpLuc has an emission peak at 454 nm wavelength [30], and a modified form of its catalytic subunit, NanoLuc, is frequently used in various applications. OpLuc exhibits flash-type luminescence in its native state, whereas its catalytic domain (19kOLase) produces glow luminescence [31,32].

Bacterial luminescence is slightly different from the D-luciferin or coelenterazine systems. The bacterial luciferase (Lux) is genetically encoded within the *luxCDABEG* operon [33]. LuxG, a flavin reductase, produces reduced flavin mononucleotide (FMNH_2_) using nicotinamide adenine dinucleotide phosphate (NADPH), and LuxCDE synthesizes long-chain aldehydes from fatty acids. LuxAB, the luciferase, catalyzes the oxidation of these long-chain aldehydes to produce excited state intermediates in the presence of FMNH_2_ and oxygen. The excited state intermediate molecules emit light around 490 nm as they relax to their ground states. Unlike other luciferases, the bacterial luminescence system is self-sufficient. All substrates are recycled and produced by enzymes encoded by the operon [34]. Thus, exogenous administration of luciferin is unnecessary for bioluminescence, making it beneficial for theranostic applications. However, introducing bacterial luciferase into eukaryotic cells requires substantial optimization efforts due to differences in cellular environments.

Taken together, each luciferase possesses unique characteristics, as summarized in Table 1, and it is crucial to select the appropriate luciferin–luciferase pair for specific applications. Furthermore, recent efforts have focused on enhancing the natural characteristics of these bioluminescence systems through the exploration of diverse luciferin analogs and mutant luciferases. Various properties such as the emission wavelength, quantum yield, kinetics, and stability of bioluminescence systems have been engineered via synthetic chemistry and protein engineering [35,36,37,38]. For example, Pozzo et al. improved the thermostability and catalytic activity of FLuc through targeted amino acid mutations [35]. In addition, Colee et al. identified specific amino acid positions on FLuc for red-shifting mutation, which can be potentially beneficial for deep tissue imaging applications [36]. The optical properties of bioluminescence, including the emission wavelength, of the same native FLuc can be adjusted by using luciferin analogs. Jathoul et al. achieved the emission spectrum of native FLuc in the near-infrared (NIR) range ($\lambda_{max}=706\;nm$) and high brightness feasible for bioluminescence imaging by developing a new luciferin analog [37]. Hall et al. engineered NanoLuc through amino acid substitution and introduced a novel imidazopyrazinone substrate. The modifications resulted in enhanced structural stability and bright luminescence, which can lead to increased sensitivity of the bioluminescence system in bioimaging and biosensing applications [39].

### 2.2. Luciferase and Luciferin Delivery

**Figure 2:**
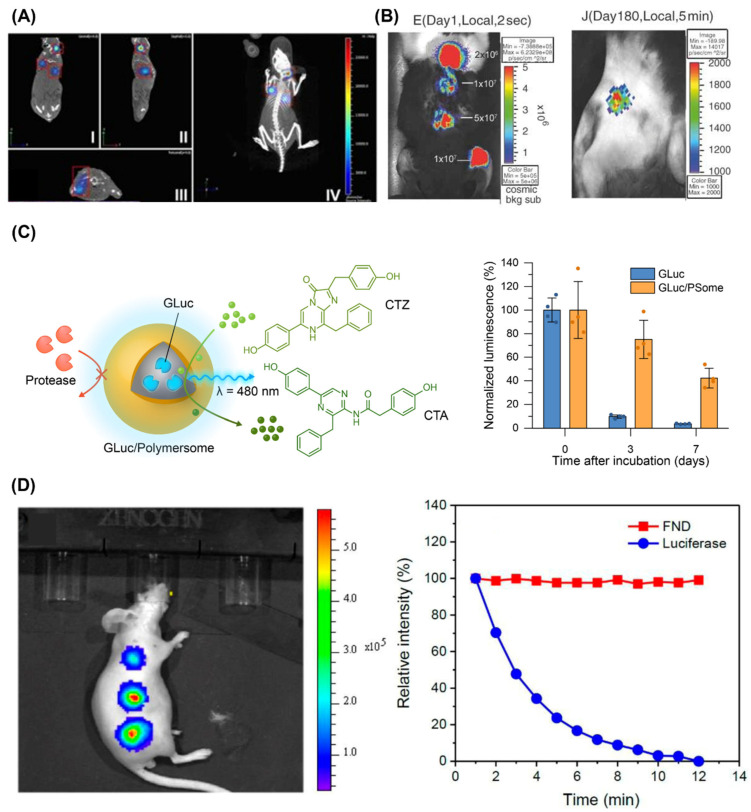
(**A**) Orthotopic breast cancer model established using luciferase-expressing cancer cells visualized by micro-CT and 3-dimensional bioluminescence imaging [42]. Copyright 2017, V. P. Baklaushev et al. (**B**) Bioluminescence images on day 1 (left) and day 180 (right) after the subcutaneous injection of a FLuc AAV vector [49]. Copyright 2005 John Wiley & Sons, Ltd. (**C**) Schematic illustration of GLuc-loaded polymersome (GLuc/PSome) and its long-term luminescence after uptake into cells [56]. Copyright 2022 H. Kim et al., Wiley-VCH. (**D**) Bioluminescence imaging of a mouse subcutaneously injected with stem cells labeled with FLuc-coated fluorescent nanodiamonds (FNDs). Decays of the fluorescence (FND) and bioluminescence (luciferase) intensities are also shown over time [57]. Copyright 2019 American Chemical Society. Adapted with permission from [42,49,56,57].

When using luciferase for theranostic applications, especially when it is used in vivo, the luciferase should be delivered to the target site of the application. The efficient delivery of luciferases is necessary to achieve the improved efficiency of the bioluminescence system. There are three main approaches that we can consider. First, we can transduce cells with the gene encoding the luciferase to achieve its stable expression and deliver the genetically modified cells to the target site. It is the most common method for bioluminescence imaging [40,41,42,43,44] and, particularly, for the development of tumor models and noninvasive monitoring of the tumor size (Figure 2A) [42]. Viral infection methods, such as lentiviral transduction and adeno-associated virus (AAV) transduction, have been optimized for efficient gene delivery [42,43,44], and nonviral vectors, such as cationic liposomes, have also been frequently used [45]. Viral infection methods provide high gene delivery efficiency, but there is still a risk of unintended genetic modifications to host cells and potential immune responses [46]. In contrast, nonviral vectors are generally considered safer but less effective than viral vectors [47]. Therefore, viral vectors are favored when high efficiency is crucial, whereas nonviral vectors are preferred when prioritizing safety.

Second, we can directly deliver genes encoding the luciferase to target tissues in vivo using either viral [48,49,50] or nonviral [51,52,53] vectors. Delivery routes and sites can be selected depending on the application, including local injections, systemic injections, and noninvasive delivery such as nasal delivery. The duration of the luciferase expression is dependent on the delivery methods, but numerous studies reported that the expression is stable over months. Li et al. administered a FLuc AAV vector via subcutaneous injection, and the luciferase expression was retained for as long as 6 months in rabbits after the injection (Figure 2B) [49]. In the case of GLuc, after the transduction and protein expression, it can be secreted into the blood. El-Amouri et al. carried out three-dimensional bioluminescence imaging after hydrodynamic tail-vein injection of GLuc-encoding plasmids and observed that the kidney/bladder, stomach/intestine, and lung are major uptake organs of GLuc [53].

Finally, we can deliver recombinant luciferase protein molecules to the target cells or tissues. It can be a simpler alternative to genetic modification which is affected by cell types and species. However, the stability of luciferase is relatively limited when delivered recombinantly than through stable genetic modification. Recombinant luciferase is susceptible to biodegradation, including thermal and proteolytic degradation, like other proteins. Therefore, a protein delivery system that can protect luciferase from the external environment while facilitating the efficient exchange of the substrate to maintain its activity is essential. Several kinds of protein delivery systems have been explored for luciferase delivery. Vesicles like liposomes [54] and polymersomes [55,56] have been employed because of their advantages as a biocompatible drug delivery system as well as unique semipermeable membrane properties. Polymersomes synthesized by photoinitiated polymerization-induced self-assembly (PISA) demonstrate luminescence production while exhibiting significant resistance to thermal, proteolytic, and intracellular stress, confirming the semipermeable nature of polymersomes (Figure 2C) [56]. Nanoparticulate systems, such as fluorescent nanodiamonds, have also been utilized as luciferase delivery platforms [57]. The fluorescent nanodiamond system enables the intracellular delivery of luciferase, allowing bioluminescence imaging and tracking of stem cells in vitro and in vivo (Figure 2D).

Luciferin delivery is also important for achieving improved bioluminescence, and various methods have been developed for the stable and targeted delivery of luciferin. For instance, liposomes have been used for delivery to protect luciferin from metabolism, reduce its toxicity, and prolong its blood circulation [58]. Luciferin encapsulated within a polyethylene glycol (PEG)-incorporated liposomal system showed a prolonged release over 24 h, while free luciferin exhibited rapid clearance in vivo (half-life of 3.54 min) [59]. Moreover, a mitotropic nanocarrier based on an oligomycin scaffold resulted in mitochondrial specificity and a significantly extended steady-state temporal profile of D-luciferin [60]. Conjugating polymers to luciferin can also extend its short circulatory half-life. PEGylation of D-luciferin, for example, led to an extension of its in vivo circulatory half-life and longer bioluminescence emission compared to free luciferin during imaging of luciferase-expressing PC3 prostate tumors in mice [61].

In addition, the administration route of luciferin is crucial for bioluminescence applications. Intraperitoneal (IP) injection of luciferin offers convenience, but its biodistribution can favor accumulation in specific organs such as the pancreas and spleen while reducing the bioluminescence signal in target tissues [62,63]. Alternatively, subcutaneous (SC) injection showed more consistent results for luciferase-expressing tissue imaging compared to IP injection [64]. The optimal administration route depends on the target tissue. For example, intranasal delivery of luciferin demonstrated its high efficiency for bioimaging of the lung and nasal cavity [65].

### 2.3. Bioluminescence Resonance Energy Transfer (BRET)

**Figure 3:**
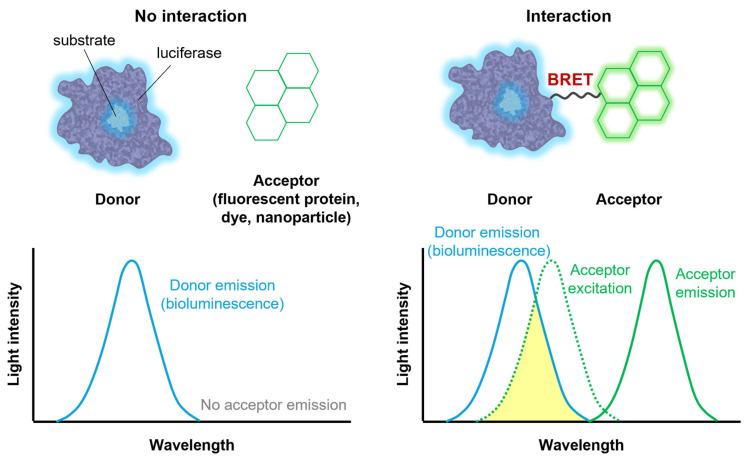
Schematic illustration of bioluminescence resonance energy transfer (BRET).

While luciferases can generate light on their own, they can also be combined with other fluorescent agents. When a bioluminescence donor and a fluorescence acceptor are in close proximity, energy transfer occurs between them, a phenomenon known as bioluminescence resonance energy transfer (BRET) [66]. BRET involves the non-radiative transfer of energy from the donor to the acceptor, occurring only when they are within a permissive distance and proper orientation, typically less than 10 nm (Figure 3). The efficiency of BRET is inversely proportional to the sixth power of the distance between the donor and acceptor.

BRET offers several advantages for biomedical applications. First, as the fluorescence acceptor is excited by energy transferred from the bioluminescence donor, the emission wavelength of the acceptor is typically longer than that of the donor. Consequently, hybrid systems can emit longer-wavelength light than the original bioluminescence [67,68]. Furthermore, because BRET efficiency depends on proximity, it can be utilized to estimate distances between interacting biomolecules. For example, BRET has been used to characterize protein–protein interactions and conformational changes within proteins or protein complexes [69,70].

The development of BRET systems involves conjugating luciferases with various fluorescent agents. Early BRET systems mainly focused on coupling luciferases with fluorescent proteins exhibiting longer emission wavelengths, such as yellow fluorescent proteins (YFP), red fluorescent proteins (RFP), and their variants [71,72]. Nano-lantern, a fusion protein combining RLuc and YFP, is a representative example of a protein BRET system, and it can generate a bright yellowish-green luminescence [73]. The color palette of Nano-lanterns has been further expanded to include cyan and orange variants by employing alternative fluorescent proteins [74]. More recently, a broader range of fluorescent agents, including fluorescent dyes [75], quantum dots [76], and other fluorescent nanoparticles [77], has been employed to expand BRET applications, such as bioimaging, biosensing, and therapeutic applications, as described in the following sections.

## 3. Theranostic Applications of Bioluminescence

### 3.1. Bioimaging

Bioluminescence imaging is a powerful imaging technique due to its unique advantages over other techniques. Since bioluminescence is produced by biochemical reactions, it does not require external light for photoexcitation, which can improve the signal-to-noise ratio and minimize phototoxicity. Bioluminescence imaging also offers high sensitivity, noninvasiveness, and real-time monitoring, making it a versatile and promising tool. In its early stages, bioluminescence imaging was primarily used for monitoring luciferase-expressing tumor cells. However, recent advances in the field have improved optical characteristics of bioluminescence, such as red-shifted emission wavelengths for deep tissue penetration [78,79,80,81,82,83], single-cell-level resolution [83], high brightness [20,81,83], long-term stability up to 12 months [84,85], and sensitivity to surrounding environments [78,86,87], leading to an expansion in its applications.

**Figure 4:**
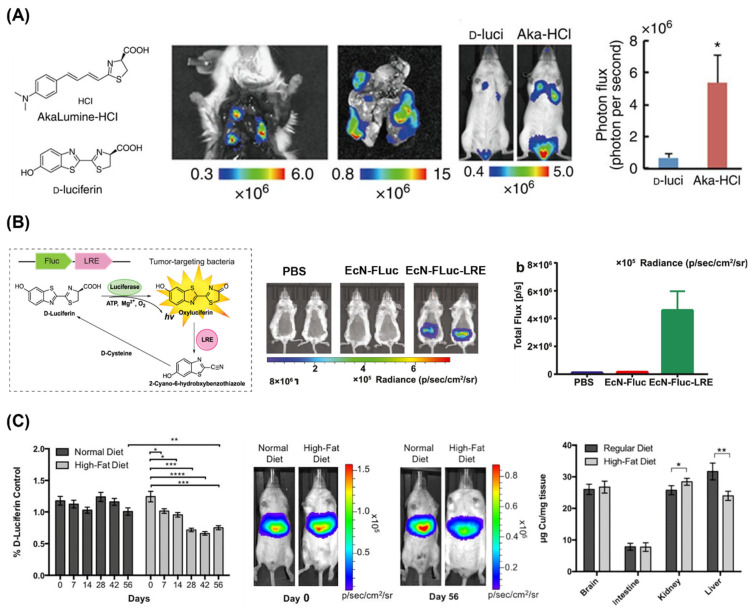
(**A**) Chemical structure of AkaLumine-HCl (Aka-HCl) and D-luciferin (D-luci) (left). Aka-HCl was used for ex vivo and in vivo bioluminescence imaging of deep tissue tumors (center). Quantitative analysis of bioluminescence shows stronger signal of Aka-HCl than D-luci (right) (* *p* < 0.05 (t-test)) [82]. Copyright 2016, T. Kuchimaru et al. (**B**) Schematic illustration of FLuc-luciferin-regenerating enzyme (LRE)-introduced bacteria (EcN) for stable bioluminescence imaging (left). EcN-FLuc-LRE was used for in vivo bioluminescence imaging of the mouse intestinal tract (center). Quantitative analysis of bioluminescence shows high signal of EcN-FLuc-LRE (right) [85]. Copyright 2021, T. Jiang et al. (**C**) Bioluminescence imaging of hepatic copper deficiency using copper-caged luciferin-1 (CCL-1) in a diet-induced murine model of nonalcoholic fatty liver disease. Bioluminescence signal was obtained after CCL-1 treatment as shown in the quantitative analysis (left) and representative images (center), and it was compared with hepatic copper levels measured by ICP-MS (right) (* *p* < 0.05, ** *p* < 0.01, *** *p* < 0.001 and **** *p* < 0.0001 (two-tailed Student’s t-test)) [87]. Copyright 2016, M. C. Heffern et al. Adapted with permission from [82,85,87].

As mentioned earlier, the emission wavelengths can be shifted to longer wavelengths, up to NIR or even NIR-II, by tuning luciferases [36,79] and luciferins [37,79,80,81,82,83] and using BRET technologies [67,68,78], and it is highly beneficial for bioluminescence imaging of deep tissues. Kuchimaru et al. synthesized a luciferin analog called AkaLumine-HCl and achieved noninvasive bioluminescence imaging of deep tissue tumors (Figure 4A) [82]. The bioluminescence produced by the reaction of AkaLumine-HCl with FLuc exhibited a maximum wavelength at 677 nm and achieved high sensitivity from deep tissues even at very low concentrations. Bioluminescence imaging using AkaLumine-HCl could visualize lung metastases ex vivo and in vivo, and AkaLumine-HCl exhibited an 8.1-fold higher signal than D-luciferin.

While early studies primarily focused on tracking cancer cell lines, recent works report bioluminescence imaging of various biological entities, including stem cells [88,89], bacteria [85,90], viruses [91,92], and extracellular vesicles [93,94]. Jiang et al. monitored the probiotic strain *Escherichia coli* Nissle 1917 (EcN) using FLuc (Figure 4B) [85]. Interestingly, they used luciferin-regenerating enzyme (LRE) together with FLuc, and LRE improved the stability of bioluminescence systems by recycling luciferin. They performed the in vivo imaging of bacteria in the mouse intestinal tract and successfully tracked the bacterial distribution. Furthermore, using LRE, they achieved a 29-fold higher bioluminescence intensity compared to normal FLuc, demonstrating the potential of this system as a tool for studying the intestinal tract.

More recently, bioluminescence imaging has been used to visualize biological phenomena beyond merely showing the location and number of biological entities [78,86,87]. Heffern et al. monitored hepatic copper deficiency by in vivo bioluminescence imaging in a murine model of nonalcoholic fatty liver disease (Figure 4C) [87]. They synthesized copper-caged luciferin-1 (CCL-1), which releases D-luciferin upon oxidative cleavage with Cu^+^ and produces bioluminescence. After feeding mice a high-fat diet to establish a nonalcoholic fatty liver disease model, they observed a significant decrease in the CCL-1 signal normalized to the D-luciferin signal, reflecting hepatic copper deficiency in response to the diet. These bioluminescence imaging results were consistent with copper levels measured by inductively coupled plasma mass spectrometry (ICP-MS). Such strategies demonstrate the potential of analyte-sensitive and biochemical environment-sensitive bioluminescence, which can be expanded to a wide variety of applications.

### 3.2. Biosensors

Bioluminescence-based biosensors offer several advantages over fluorescence-based biosensors and have been investigated for detecting various analytes. Since bioluminescence does not require an external light source, it can avoid issues related to background signal and photobleaching, resulting in a high signal-to-noise ratio. Moreover, bioluminescence can provide a continuous signal as long as the substrate presents, enabling real-time monitoring of biological processes. Flexibility in the design of bioluminescent systems also provides a dynamic range of signals, versatility in molecular interactions, and improved sensitivity and specificity [95]. For example, the use of luciferase and luciferin with increased bioluminescence production can significantly amplify weak signals, enhancing the sensitivity of the system [82]. Therefore, a variety of biosensor systems utilizing bioluminescence have been developed. Many of these systems are based on BRET discussed in Section 2.3, but other mechanisms involving analyte-sensitive substrates, luciferases, and linkers have also been explored.

**Figure 5:**
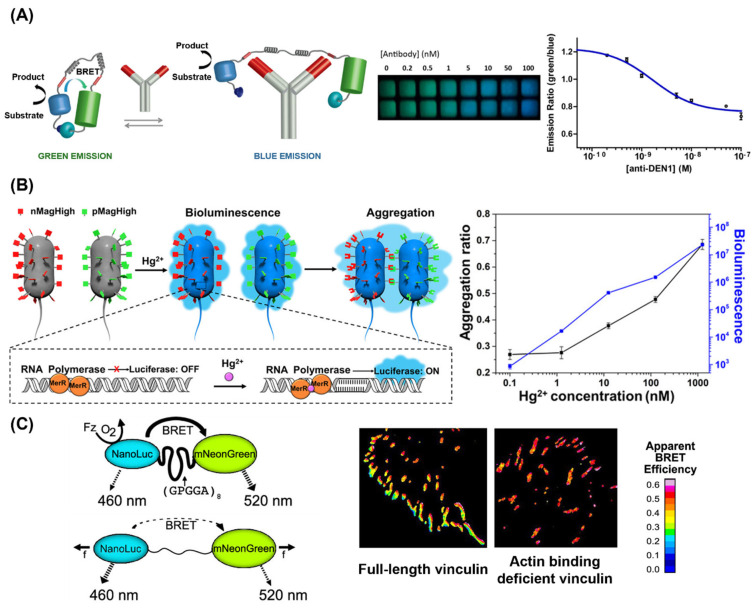
(**A**) Schematic representation of the BRET sensor for detecting antibodies (left) and the results obtained using a smartphone for the titration of the antibody against dengue virus type I (anti-DEN1) in a buffer (right) [97]. Copyright 2016 American Chemical Society. (**B**) Schematic representation of the bioluminescent bacterial sensor for detecting Hg^2+^ (left) and the dose-dependent results of bacterial aggregation ratio and bioluminescence (right) [109]. Copyright 2020 American Chemical Society. (**C**) Schematic representation of the BRET molecular tension sensor (left) and results with full-length vinculin and actin-binding-deficient vinculin (right) [111]. Copyright 2019 American Chemical Society. Adapted with permission from [97,109,111].

One of the earliest and simplest bioluminescence-based sensor systems was designed for counting live cells and evaluating the cytotoxicity of samples based on the ATP dependence of FLuc bioluminescence [96]. Subsequently, biosensors for detecting specific agents in complex biological samples were developed. Proteins, including antibodies, have been studied as analytes for biosensor systems due to their crucial roles in biological processes and their unique interactions with biological molecules [97,98,99,100,101,102]. Arts et al. developed a bioluminescent sensor for detecting antibodies in blood plasma based on BRET (Figure 5A) [97]. They designed the BRET system with a blue light-emitting luciferase connected via a semiflexible linker to a green fluorescent acceptor protein. Binding of the target antibody to the linker decreases BRET efficiency, which can be observed as a color change from green to blue. The system demonstrated high sensitivity compared to previously reported antibody detection assays, reporting a detection limit of 10 pM due to the high brightness of NanoLuc. Moreover, this detection could be easily performed using a smartphone, and the target specificity could be easily modified to detect another antibody by changing the epitope sequences. Building on these studies, recent approaches focus on analyzing protein activity, not just concentration, enabling various types of assays to monitor protein dynamics [103,104,105].

While many studies focus on detecting proteins, bioluminescent sensors can also detect small molecules. Griss et al. designed a BRET sensor that can selectively recognize a wide range of drugs, such as anticancer agents, immunosuppressants, antiepileptics, and antiarrhythmics [106]. For example, to target the anticancer agent methotrexate, they incorporated dihydrofolate reductase (DHFR) as a receptor protein and DHFR inhibitors as an intramolecular ligand into a BRET system. When methotrexate was added, it bound to DHFR, disrupting the BRET system and decreasing the BRET efficiency, which allows for concentration-dependent detection of methotrexate. By changing the receptor protein, this platform can be adapted for highly specific detection of various drugs, enabling point-of-care therapeutic drug monitoring in patient samples.

Several bioluminescent sensors have been employed to detect metal ions, such as Zn^2+^ [107,108] and Hg^2+^ [109,110]. Similar to the detection of other molecules, BRET can be used for the detection, but other approaches have also been reported. Chen et al. developed a highly sensitive dual-output biosensor by employing a Hg^2+^-sensitive bioluminescence reporter combined with light-responsive bacterial aggregation (Figure 5B) [109]. Upon Hg^2+^ binding, the expression of bacterial luciferase is induced, causing the bacteria to emit blue luminescence. Subsequently, the luminescence activates the photoswitchable proteins nMagHigh and pMagHigh, leading to bacterial aggregation. Therefore, both bacterial aggregation and bioluminescence increase with elevating Hg^2+^ concentration, enhancing the sensitivity of the biosensor.

Bioluminescent sensors can also function as molecular tension sensors. Aird et al. developed a BRET-based molecular tension sensor that can measure piconewton forces (Figure 5C) [111]. In this sensor, the NanoLuc donor and mNeonGreen acceptor proteins are linked by a flexible spider silk flagelliform domain (GPGGA)_8_. When force is applied, the donor–acceptor pair separates, causing a decrease in BRET efficiency. The system was adapted to monitor the focal adhesion protein vinculin. With full-length vinculin, BRET efficiency decreased at the cell periphery, where forces are higher (peripheral focal adhesion), and gradually increased toward the nucleus. This gradient was not observed in actin-binding-deficient vinculin, which is force-insensitive. Overall, bioluminescent sensors have been developed for detecting diverse analytes and are expected to expand into a variety of applications.

### 3.3. Photodynamic Therapy

Photodynamic therapy (PDT) is a therapeutic approach that employs light to activate photosensitizers, generating reactive oxygen species (ROS) [112,113]. This method has been actively applied for the treatment of various diseases, including cancer and infectious diseases, due to its minimal invasiveness, milder side effects, and versatility. However, the low efficacy of PDT in deep tissues, resulting from limited light penetration, has hindered its further clinical applications. To address this challenge, internal light sources have been explored, with bioluminescence emerging as a particularly attractive option for PDT.

**Figure 6:**
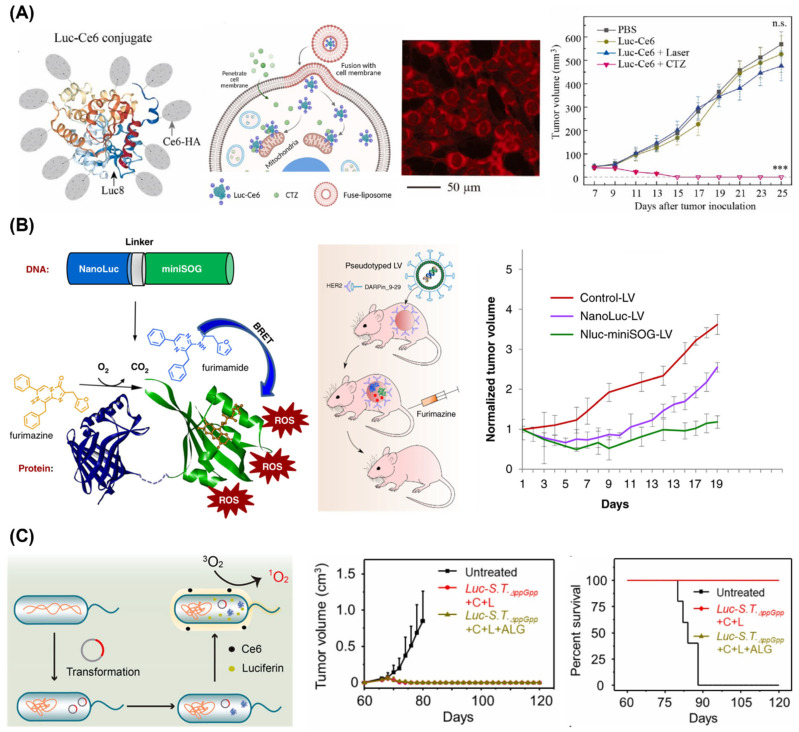
(**A**) Schematic representation of the BRET-based PDT system. Luciferase-chlorin e6 conjugates (Luc-Ce6) in membrane-fusion liposomes (left). The BRET system (red) was efficiently delivered into cytosol of 4T1 cells (center) and showed therapeutic effect on early-stage 4T1 tumor (right) (*** *p* < 0.001 and n.s.: not significant (two-way ANOVA)) [115]. Copyright 2023 Elsevier Ltd. (**B**) Schematic representations of the gene encoding NanoLuc luciferase fused to miniSOG phototoxic protein and its lentiviral delivery to HER-2 positive tumor (left). The lentiviral delivery of NanoLuc-miniSOG inhibited the tumor growth (right) [126]. Copyright 2022, E. I. Shramova et al. (**C**) Schematic representation of bioluminescent bacteria ($Luc-S.T._{∆ppGpp}$) (left) and its PDT effect on inhibition of tumor metastasis and recurrence (right). Tumor growth curves and survival rate of rechallenged tumor 60 days post-treatment are represented (C: Ce6, L: luciferin, and ALG: alginate hydrogel) [127]. Copyright 2021 Elsevier Ltd. Adapted with permission from [115,126,127].

Although bioluminescence is much weaker than conventional light sources like lasers and LEDs, efficient BRET from bioluminescent systems to photosensitizers can significantly enhance PDT efficacy [114,115,116,117,118,119]. Various BRET systems have been developed, including those using chemical conjugation [114,115,116,117] and luciferase-ROS generating protein fusion proteins [118,119]. When the emission of bioluminescent systems does not match the photosensitizer, fluorescent nanoparticles, such as quantum dots [120,121] and carbon dots [122], can be additionally introduced to mediate efficient PDT. The therapeutic efficacy of these BRET systems can be further improved by using appropriate nanocarrier systems [114,115,119]. Yan et al. developed a luciferase-photosensitizer (chlorin e6, Ce6) conjugate and achieved its intracellular delivery using membrane-fusion liposomes (Figure 6A) [115]. The conjugates resulted in effective and targeted cancer cell killing in an orthotopic mouse model of 4T1 triple-negative breast cancer, leading to complete tumor remission and prevention of metastasis for early-stage tumors.

To improve the intracellular delivery of luciferases, gene delivery methods have been considered for PDT [123,124,125,126]. Gene delivery using polymer particles or viral vectors can produce high concentrations of luciferases in the cells, facilitating efficient PDT. Shramova et al. developed genes encoding a BRET pair, NanoLuc luciferase and phototoxic flavoprotein miniSOG, and delivered it into tumor tissues expressing human epidermal growth receptor 2 (HER2) in vivo using tumor-specific lentiviral particles (Figure 6B) [126]. This target-specific delivery of the BRET gene resulted in successful deep tissue PDT and inhibited tumor growth.

Bioluminescent bacteria can also be used for PDT [127]. Yang et al. engineered bioluminescent bacteria by introducing a FLuc-expressing plasmid into an attenuated *Salmonella typhimurium* strain ∆ppGpp (${S.T.}_{∆ppGpp}$) and delivered it into tumor tissues using in-situ-formed alginate hydrogel (Figure 6C). The bioluminescent bacteria generated light upon luciferin addition, activating Ce6 for PDT. The activated Ce6 can facilitate the transfer of energy to oxygen to produce ROS, leading to apoptosis and/or necrosis in tumor cells and effective suppression of tumor growth. Remarkably, the bioluminescent bacteria-mediated PDT not only suppressed tumor growth but also elicited a potent antitumor immune response, inhibiting tumor metastasis and preventing tumor recurrence, showing its potential for clinical translation. As various bioluminescent systems continue to be optimized and improved, their clinical applications in PDT are anticipated in the near future.

### 3.4. Optogenetic Applications

**Figure 7:**
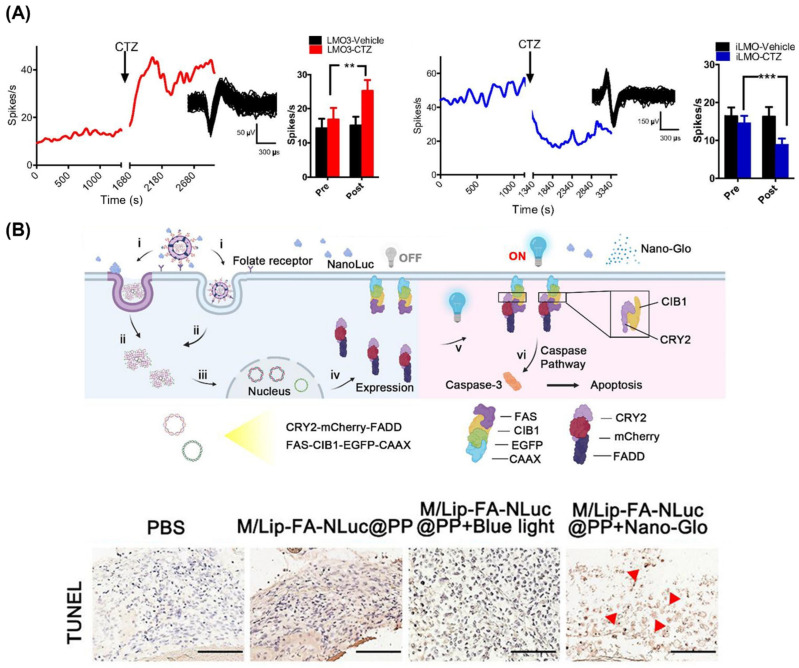
(**A**) Virus-mediated expression of bioluminescent optogenetic systems (LMO3 or iLMO) modulates neuronal firing rates in vivo, inducing increases (left) or decreases (right) upon the addition of coelenterazine (CTZ) (** *p* < 0.01 and *** *p* < 0.001 (two-way ANOVA followed by post hoc test)) [131]. Copyright 2016, K. Berglund et al. (**B**) Schematic representation of in situ bioluminescence-driven optogenetic therapy of retinoblastoma based on camouflage nanoparticles (top). The delivery of optogenetic plasmids results in the expression of FAS-CIB1-EGFP-CAAX in the plasma membrane and the expression of CRY2-mCherry-FADD in the cytoplasm. Blue bioluminescence induces the binding of the blue light receptor proteins CIB1 and CRY2, and the subsequent binding of FADD to FAS activates the caspase-3 apoptotic signaling pathway. In vivo evaluation of camouflage nanoparticle-based optogenetic system demonstrated more significant tumor cell apoptosis upon addition of Nano-Glo (furimazine) than in other groups [141]. Red arrows indicate regions of tumor cell apoptosis. Scale bars, 100 μm. Copyright 2023 American Chemical Society. Adapted with permission from [131,141].

Optogenetic approaches were initiated by transgenic modification of mammalian cells to express light-activated ion channels, allowing for light-based modulation of cellular functions such as neuronal activity and cardiomyocyte contraction [128,129]. Since the fusion of luciferase to a light-activated ion channel for optogenetic applications was first reported [130], bioluminescent optogenetics has been actively investigated as a noninvasive tool to manipulate neuronal activity [131,132,133,134]. Berglund et al. demonstrated that a bioluminescent optogenetic system can either excite or inhibit neurons by fusing GLuc with either channelrhodopsin or a proton pump (Figure 7A) [131]. They injected viral vectors encoding these fusion proteins into the nigra pars reticulata of mice and showed that neuronal firing rates could be increased or decreased depending on the type of opsin, following coelenterazine addition. Subsequently, bioluminescent optogenetics has been continuously explored, leading to the development of systems with tuned emission wavelengths [135] and improved coupling efficiencies [136]. These systems are now being studied for the treatment of neuronal diseases, such as restoration after spinal cord injury [137], axon regeneration after peripheral nerve injury [138,139], and improvement of motor deficits in Parkinson’s disease [140].

Bioluminescent optogenetic approaches are now expanding into numerous applications. Ding et al. reported the use of camouflage nanoparticles for bioluminescence-driven optogenetic therapy of retinoblastoma (Figure 7B) [141]. These nanoparticles delivered NanoLuc luciferase along with optogenetic plasmids, expressing proteins that activate the caspase-3-mediated apoptotic pathway to inhibit tumor growth upon bioluminescence. In vivo application of this system for retinoblastoma treatment demonstrated the promotion of apoptosis and inhibition of tumor growth while reducing the phototoxic and photodamaging effects of light on tissues. Moreover, bioluminescent optogenetic systems have been utilized for modulating cardiomyocyte contraction [56], facilitating protein communication within synthetic cells [142] and inducing necroptotic and pyroptotic cell death in living animals [143], proving their versatility.

While bioluminescence offers a minimally invasive approach to optogenetics as an internal light source, its spatiotemporal resolution is limited compared to LEDs or lasers, which can restrict the applicability of bioluminescence in certain optogenetic applications. In addition, pulsed light from conventional optogenetic devices often elicits distinct cellular responses compared to the sustained light produced by bioluminescence. For example, pulsed light can induce light-synchronized calcium spikes and contractions in cardiomyocytes, while continuous light from LEDs or bioluminescence simply increases the spontaneous beating rate [56]. However, since many in vivo applications often do not require the highest temporal resolution [131], the bioluminescent optogenetic approach remains a valuable strategy to facilitate safe light delivery into deep tissues and minimize photodamage.

## 4. Summary and Perspectives

Bioluminescent systems have emerged as promising tools for light-based theranostic applications, offering advantages as internal light sources, particularly for applications requiring deep tissue penetration. Despite remarkable progress in expanding their emission wavelengths, kinetics, and overall efficiency, the limited stability and intensity of bioluminescence remain significant hurdles for broader adoption. Because bioluminescence is derived from enzymatic reactions, its stability is inherently limited, especially in vivo. The intensity of bioluminescence varies with the concentration and types of luciferases and luciferin, but it is generally weaker than other electronic light sources commonly used in theranostics, such as LEDs and lasers. In addition, the activity of enzymes is sensitive to environmental factors, including pH and temperature, further affecting the stability and intensity of bioluminescence [144].

To address these challenges, numerous strategies are being developed, including the stable expression of luciferase through gene delivery techniques, the development of long-term delivery carriers for sustained luciferase delivery, and the use of BRET-based techniques, as discussed in the previous sections. Moreover, the development of mutants of luciferase has been considered a promising approach to enhance both the activity and stability of luciferases. Recently, inspired by advancements in artificial intelligence (AI), designing luciferase sequences based on generative models has attracted attention in the field. Initial trials have demonstrated that AI-based luciferase engineering can significantly improve luciferase activity and stability [145,146]. The technique can be also employed for diversifying biochemical and optical characteristics of bioluminescent systems, which is crucial for advancing bioluminescent systems in theranostics. On the other hand, in the case of luciferase/luciferin delivery carriers, strategies to modulate the enzyme’s surrounding environment, such as pH control, are important for optimizing bioluminescence production beyond simply protecting luciferases from degradation.

It is important to note that advancements in light-based diagnostics and therapeutics lead the development of novel bioluminescent systems. For example, progress in phototherapy employing low-intensity light, such as low-level laser therapy (LLLT), can promote theranostic applications of bioluminescent systems. While LLLT has been mainly employed for skin due to the limited penetration of light into deep tissues [2,147], bioluminescence holds potential for photobiomodulation in deep tissues like the brain or nerves. As light-based diagnostic and therapeutic approaches expand, the significance of bioluminescent systems is expected to increase in future clinical settings.

## Figures and Tables

**Table 1:** Representative luciferins and luciferases used for theranostic applications.

Luciferins	Luciferases (Molecular Weight)	Requirements for Reactions	$\mathrm{Wavelength}\;(\lambda_{max}$, nm)	Kinetics
D-luciferin	Firefly luciferase (62 kDa)	ATP, Mg^2+^, O_2_	558	Glow
Coelenterazine	*Renilla* luciferase (36 kDa)	O_2_	480	Flash
	*Gaussia* luciferase (20 kDa)	O_2_	485	Flash
	*Oplophorus* luciferase (106 kDa)	O_2_	454	Flash
Long-chain aldehyde	Bacterial luciferase (77 kDa)	FMNH_2_, O_2_	490	Flash

ATP: adenyl triphosphate; FMNH_2_: reduced flavin mononucleotide.

## References

[1] Yun S.H., Kwok S.J.J. (2017). Light in diagnosis, therapy and surgery. Nat. Biomed. Eng..

[2] Lee G.-H., Moon H., Kim H., Lee G.H., Kwon W., Yoo S., Myung D., Yun S.H., Bao Z., Hahn S.K. (2020). Multifunctional materials for implantable and wearable photonic healthcare devices. Nat. Rev. Mater..

[3] Wolfbeis O.S. (2015). An overview of nanoparticles commonly used in fluorescent bioimaging. Chem. Soc. Rev..

[4] Wang S., Li B., Zhang F. (2020). Molecular fluorophores for deep-tissue bioimaging. ACS Central. Sci..

[5] Liu S., Pan X., Liu H. (2020). Two-dimensional nanomaterials for photothermal therapy. Angew. Chem. Int. Ed..

[6] Pham T.C., Nguyen V.-N., Choi Y., Lee S., Yoon J. (2021). Recent strategies to develop innovative photosensitizers for enhanced photodynamic therapy. Chem. Rev..

[7] de Freitas L.F., Hamblin M.R. (2016). Proposed mechanisms of photobiomodulation or low-level light therapy. IEEE J. Sel. Top. Quantum Electron..

[8] Wang M., Rao J., Wang M., Li X., Liu K., Naylor M.F., Nordquist R.E., Chen W.R., Zhou F. (2021). Cancer photo-immunotherapy: From bench to bedside. Theranostics.

[9] Chen D., Tang Y., Zhu J., Zhang J., Song X., Wang W., Shao J., Huang W., Chen P., Dong X. (2019). Photothermal-pH-hypoxia responsive multifunctional nanoplatform for cancer photo-chemo therapy with negligible skin phototoxicity. Biomaterials.

[10] Arany P.R., Cho A., Hunt T.D., Sidhu G., Shin K., Hahm E., Huang G.X., Weaver J., Chen A.C.-H., Padwa B.L. (2014). Photoactivation of endogenous latent transforming growth factor-β1 directs dental stem cell differentiation for regeneration. Sci. Transl. Med..

[11] Peng J., Zhao J., Tang Q., Wang J., Song W., Lu X., Huang X., Chen G., Zheng W., Zhang L. (2022). Low intensity near-infrared light promotes bone regeneration via circadian clock protein cryptochrome 1. Int. J. Oral Sci..

[12] Sordillo L.A., Pu Y., Pratavieria S., Budansky Y., Alfano R.R. (2014). Deep optical imaging of tissue using the second and third near-infrared spectral windows. J. Biomed. Opt..

[13] Choi J., Lee I.S., Lee J.S., Jeon S., Yun W.S., Yang S., Moon Y., Kim J., Kim J., Choy S. (2022). Implantable micro-scale LED device guided photodynamic therapy to potentiate antitumor immunity with mild visible light. Biomater. Res..

[14] Ran Y., Xu Z., Chen M., Wang W., Wu Y., Cai J., Long J., Chen Z.-S., Zhang D., Guan B.-Q. (2022). Fiber-optic theranostics (FOT): Interstitial fiber-optic needles for cancer sensing and therapy. Adv. Sci..

[15] Wang D., Kuzma M.L., Tan X., He T.-C., Dong C., Liu Z., Yang J. (2021). Phototherapy and optical waveguides for the treatment of infection. Adv. Drug Deliv. Rev..

[16] Wilson T., Hastings J.W. (1998). Bioluminescence. Annu. Rev. Cell Dev. Biol..

[17] Haddock S.H.D., Moline M.A., Case J.F. (2010). Bioluminescence in the sea. Annu. Rev. Mar. Sci..

[18] Alsawaftah N., Farooq A., Dhou S., Majdalawieh A.F. (2021). Bioluminescence imaging applications in cancer: A comprehensive review. IEEE Rev. Biomed. Eng..

[19] Yang M., Huang J., Fan J., Du J., Pu K., Peng X. (2020). Chemiluminescence for bioimaging and therapeutics: Recent advances and challenges. Chem. Soc. Rev..

[20] Su Y., Walker J.R., Park Y., Smith T.P., Liu L.X., Hall M.P., Labanieh L., Hurst R., Wang D.C., Encell L.P. (2020). Novel NanoLuc substrates enable bright two-population bioluminescence imaging in animals. Nat. Methods.

[21] Syed A.J., Anderson J.C. (2021). Applications of bioluminescence in biotechnology and beyond. Chem. Soc. Rev..

[22] Adams S.T., Miller S.C. (2020). Enzymatic promiscuity and the evolution of bioluminescence. FEBS J..

[23] Liu S., Su Y., Lin M.Z., Ronald J.A. (2021). Brightening up biology: Advances in luciferase systems for in vivo imaging. ACS Chem. Biol..

[24] Li S., Ruan Z., Zhang H., Xu H. (2021). Recent achievements of bioluminescence imaging based on firefly luciferin-luciferase system. Eur. J. Med. Chem..

[25] Ando Y., Niwa K., Yamada N., Enomoto T., Irie T., Kubota H., Ohmiya Y., Akiyama H. (2008). Firefly bioluminescence quantum yield and colour change by pH-sensitive green emission. Nat. Photon..

[26] Morse D., Tannous B.A. (2012). A water-soluble coelenterazine for sensitive in vivo imaging of coelenterate luciferases. Mol. Ther..

[27] Jiang T., Du L., Li M. (2016). Lighting up bioluminescence with coelenterazine: Strategies and applications. Photochem. Photobiol. Sci..

[28] Markova S.V., Larionova M.D., Vysotski E.S. (2019). Shining light on the secreted luciferases of marine copepods: Current knowledge and applications. Photochem. Photobiol..

[29] Hunt E.A., Moutsiopoulou A., Ioannou S., Ahern K., Woodward K., Dikici E., Daunert S., Deo S.K. (2016). Truncated variants of Gaussia luciferase with tyrosine linker for site-specific bioconjugate applications. Sci. Rep..

[30] Nakamura H., Wu C., Murai A., Inouye S., Shimomura O. (1997). Efficient bioluminescence of bisdeoxycoelenterazine with the luciferase of a deep-sea shrimp OpZophorus. Tetrahedron Lett..

[31] Inouye S., Sasaki S. (2007). Overexpression, purification and characterization of the catalytic component of Oplophorus luciferase in the deep-sea shrimp, Oplophorus gracilirostris. Protein Expr. Purif..

[32] Inouye S., Sahara-Miura Y., Sato J., Iimori R., Yosida S., Hosoya T. (2013). Expression, purification and luminescence properties of coelenterazine-utilizing luciferases from Renilla, Oplophorus and Gaussia: Comparison of substrate specificity for C2-modified coelenterazines. Protein Expr. Purif..

[33] Close D., Xu T., Smartt A., Rogers A., Crossley R., Price S., Ripp S., Sayler G. (2012). The evolution of bacterial luciferase gene cassette (lux) as a real-time reporter. Sensors.

[34] Tinikul R., Chunthaboon P., Phonbuppha J., Paladkong T. (2020). Chapter fourteen—Bacterial luciferase: Molecular mechanisms and applications. Enzymes.

[35] Pozzo T., Akter F., Nomura Y., Louie A.Y., Yokobayashi Y. (2018). Firefly luciferase mutant with enhanced activity and thermostability. ACS Omega.

[36] Colee C.M., Oberlag N.M., Simon M., Chapman O.S., Flanagan L.C., Reid-McLaughlin E.S., Gewing-Mullins J.A., Maiche S., Patel D.F., Cavalcanti A.R.O. (2024). Discovery of red-shifting mutations in firefly luciferase using high-throughput biochemistry. Biochemistry.

[37] Jathoul A.P., Grounds H., Anderson J.C., Pule M.A. (2014). A dual-color far-red to near-infrared firefly luciferin analogue designed for multiparametric bioluminescence imaging. Angew. Chem. Int. Ed..

[38] Gupta R., Kasturi P., Bracher A., Loew C., Zheng M., Villella A., Garza D., Hartl F.U., Raychaudhuri S. (2011). Firefly luciferase mutants as sensors of proteome stress. Nat. Methods.

[39] Hall M.P., Unch J., Binkowski B.F., Valley M.P., Butler B.L., Wood M.G., Otto P., Zimmerman K., Vidugiris G., Machleidt T. (2012). Engineered luciferase reporter from a deep sea shrimp utilizing a novel imidazopyrazinone substrate. ACS Chem. Biol..

[40] Bhaumik S., Gambhir S.S. (2002). Optical imaging of Renilla luciferase reporter gene expression in living mice. Proc. Natl. Acad Sci. USA.

[41] Badr C.E., Tannous B.A. (2011). Bioluminescence imaging: Progress and applications. Trends Biotechnol..

[42] Baklaushev V.P., Kilpeläinen A., Petkov S., Abakumov M.A., Grinenko N.F., Yusubalieva G.M., Latanova A.A., Gubskiy I.L., Zabozlaev F.G., Starodubova E.S. (2017). Bioluminescence imaging but imposes limitations on the orthotopic mouse (4T1) model of breast cancer. Sci. Rep..

[43] Brennan T.V., Lin L., Huang X., Yang Y. (2018). Generation of luciferase-expressing tumor cell lines. Bio-Protocol.

[44] Niu G., Xiong Z., Cheng Z., Cai W., Gambhir S.S., Xing L., Chen X. (2007). In vivo bioluminescence tumor imaging of RGD peptide-modified adenoviral vector encoding firefly luciferase reporter gene. Mol. Imaging Biol..

[45] Yamano S., Dai J., Moursi A.M. (2010). Comparison of transfection efficiency of nonviral gene transfer reagents. Mol. Biotechnol..

[46] Mintzer M.A., Simanek E.E. (2009). Nonviral vectors for gene delivery. Chem. Rev..

[47] Patil S., Gao Y.-G., Lin X., Li Y., Dang K., Tian Y., Zhang W.-J., Jiang S.-F., Qadir A., Qian A.-R. (2019). The development of functional non-viral vectors for gene delivery. Int. J. Mol. Sci..

[48] Tarantal A.F., Lee C.C.I. (2010). Long-term luciferase expression monitored by bioluminescence imaging after adeno-associated virus-mediated fetal gene delivery in Rhesus monkeys (*Macaca mulatta*). Hum. Gene Ther..

[49] Li J.Z., Holman D., Li H., Liu A.-H., Beres B., Hankins G.R., Helm G.A. (2005). Long-term tracing of adenoviral expression in rat and rabbit using luciferase imaging. J. Gene Med..

[50] Tannous B.A. (2009). Gaussia luciferase reporter assay for monitoring biological processes in culture and in vivo. Nat. Protoc..

[51] Griesenbach U., Vicente C.C., Roberts M.J., Meng C., Soussi S., Xenariou S., Tennant P., Baker A., Baker E., Gordon C. (2011). Secreted Gaussia luciferase as a sensitive reporter gene for in vivo and ex vivo studies of airway gene transfer. Biomaterials.

[52] Nakanishi H., Higuchi Y., Kawakami S., Yamashita F., Hashida M. (2010). piggyBac Transposom-mediated long-term gene expression in mice. Mol. Ther..

[53] El-Amouri S.S., Cao P., Miao C. (2013). Secreted luciferase for in vivo enhancement of systemic protein delivery in mice. Mol. Biotechnol..

[54] Han X.-J., Wei Y.-F., Wan Y.-Y., Jiang L.-P., Zhang J.-F., Xin H.-B. (2014). Development of a novel liposomal nanodelivery system for bioluminescence imaging and targeted drug delivery in ErbB2-overexpressing metastatic ovarian carcinoma. Int. J. Mol. Med..

[55] Mery C.E., Craciun I., Schoenenberger C.-A., Wehr R., Palivan C.R. (2021). Catalytic polymersomes to produce strong and long-lasting bioluminescence. Nanoscale.

[56] Kim H., Yeow J., Najer A., Kit-Anan W., Wang R., Rafaie-Graham O., Thanapongpibul C., Stevens M.M. (2022). Microliter scale synthesis of luciferase-encapsulated polymersomes as artificial organelles for optogenetic modulation of cardiomyocyte beating. Adv. Sci..

[57] Su L.-J., Lin H.-H., Wu M.-S., Pan L., Yadav K., Hsu H.-H., Ling T.-Y., Chen Y.-T., Chang H.-C. (2019). Intracellular delivery of luciferase with fluorescent nanodiamonds for dual-modality imaging of human stem cells. Bioconjug. Chem..

[58] Torchilin V.P. (2005). Recent advances with liposomes as pharmaceutical carriers. Nat. Rev. Drug Discov..

[59] Kheirolomoom A., Kruse D.E., Qin S., Watson K.E., Lai C.-Y., Young L.J., Cardiff R.D., Ferrera K.W. (2010). Enhanced in vivo bioluminescence imaging using liposomal luciferin delivery system. J. Control Release.

[60] Theodossiou T.A., Sideratou Z., Tsiourvas D., Paleos C.M. (2011). A novel mitotropic oligolysine nanocarrier: Targeted delivery of covalently bound D-Luciferin to cell mitochondria. Mitochondrion.

[61] Chandran S.S., Williams S.A., Denmeade S.R. (2009). Extended-release PEG-luciferin allows for long-term imaging of firefly luciferase activity in vivo. Luminescence.

[62] Inoue Y., Kiryu S., Izawa K., Watanabe M., Tojo A., Ohtomo K. (2009). Comparison of subcutaneous and intraperitoneal injection of D-luciferin for in vivo bioluminescence imaging. Eur. J. Nucl. Med. Mol. Imaging.

[63] Lee K.-H., Byun S.S., Paik J.-Y., Lee S.Y., Song S.H., Choe Y.S., Kim B.-T. (2003). Cell uptake and tissue distribution of radioiodine labelled D-luciferin: Implications for luciferase based gene imaging. Nucl. Med. Commun..

[64] Khalil A.A., Jameson M.J., Broaddus W., Chung T.D., Golding S.E., Dever S.M., Rosenberg E., Valerie K. (2013). Subcutaneous administration of D-luciferin is an effective alternative to intraperitoneal injection in bioluminescence imaging of xenograft tumors in nude mice. ISRN Mol. Imaging.

[65] Buckley S.M., Howe S.J., Rahim A.A., Burning H., Mcintosh J., Wong S.-P., Baker A.H., Nathwani A., Thrasher A.J., Coutelle C. (2008). Luciferin detection after intranasal vector delivery is improved by intranasal rather than intraperitoneal luciferin administration. Hum. Gene Ther..

[66] Kobayashi H., Picard L.-P., Schönegge A.-M., Bouvier M. (2019). Bioluminescence resonance energy transfer-based imaging of protein-protein interactions in living cells. Nat. Protoc..

[67] Hiblot J., Yu Q., Sabbadini M.D.B., Reymond L., Xue L., Schena A., Sallin O., Hill N., Griss R., Johnsson K. (2017). Luciferases with tunable emission wavelengths. Angew. Chem. Int. Ed..

[68] Weihs F., Wang J., Pfleger K.D.G., Dacres H. (2020). Experimental determination of the bioluminescence resonance energy transfer (BRET) Förster distances of NanoBRET and red-shifted BRET pairs. Anal. Chim. Acta X.

[69] Hellweg L., Edenhofer A., Barck L., Huppertz M.-C., Frei M.S., Tarnawski M., Bergner A., Koch B., Johnsson K., Hiblot J. (2022). A general method for the development of multicolor biosensors with large dynamic ranges. Nat. Chem. Biol..

[70] Pfleger K.D.G., Eidne K.A. (2006). Illuminating insights into protein-protein interactions using bioluminescence resonance energy transfer (BRET). Nat. Methods.

[71] Breton B., Sauvageau É., Zhou J., Bonin H., Gouill C.L., Bouvier M. (2010). Multiplexing of multicolor bioluminescence resonance energy transfer. Biophys. J..

[72] Carriba P., Navarro G., Ciruela F., Ferré S., Casadó V., Agnati L., Cortés A., Mallol J., Fuxe K., Canela E.I. (2008). Detection of heteromerization of more than two proteins by sequential BRET-FRET. Nat. Methods.

[73] Saito K., Chang Y.-F., Horikawa K., Hatsugai N., Higuchi Y., Hashida M., Yoshida Y., Matsuda T., Arai Y., Nagai T. (2012). Luminescent proteins for high-speed single-cell and whole-body imaging. Nat. Commun..

[74] Takai A., Nakano M., Saito K., Haruno R., Watanabe T.M., Ohyanagi T., Jin T., Okada Y., Nagai T. (2015). Expanded palette of Nano-lanterns for real-time multicolor luminescence imaging. Proc. Natl. Acad. Sci. USA.

[75] Takahiro K., Suka T., Hirota K., Kadonosono T., Kizaka-Kondoh S. (2016). A novel injectable BRET-based in vivo imaging probe for detecting the activity of hypoxia-inducible factor regulated by the ubiquitin-proteasome system. Sci. Rep..

[76] Wu C., Kawasaki K., Ohgiya S., Ohmiya Y. (2011). Chemical studies on the BRET system between the bioluminescence of Cyprindina and quantum dots. Photochem. Photobiol. Sci..

[77] Xiong L., Shuhendler A.J., Rao J. (2012). Self-luminescing BRET-FRET near-infrared dots for in vivo lymph-node mapping and tumor imaging. Nat. Commun..

[78] Lu L., Li B., Ding S., Fan Y., Wang S., Sun C., Zhao M., Zhao C.-X., Zhang F. (2020). NIR-II bioluminescence for in vivo high contrast imaging and in situ ATP-mediated metastases tracing. Nat. Commun..

[79] Yao Z., Zhang B.S., Steinhardt R.C., Mills J.H., Prescher J.A. (2020). Multicomponent bioluminescence imaging with a π-extended luciferin. J. Am. Chem. Soc..

[80] Tamaki S., Kitada N., Kiyama M., Fujii R., Hirano T., Kim S.B., Maki S. (2021). Color-tunable bioluminescence imaging portfolio for cell imaging. Sci. Rep..

[81] Evans M.S., Chaurette J.P., Adams S.T., Reddy G.R., Paley M.A., Aronin N., Prescher J.A., Miller S.C. (2014). A synthetic luciferin improves bioluminescence imaging in live mice. Nat. Methods.

[82] Kuchimaru T., Iwano S., Kiyama M., Mitsumata S., Kadonosono T., Niwa H., Maki S., Kizaka-Kondoh S. (2016). A luciferin analogue generating near-infrared bioluminescence achieves highly sensitive deep-tissue imaging. Nat. Commun..

[83] Iwano S., Sugiyama M., Hama H., Watakabe A., Hasegawa N., Kuchimaru T., Tanaka K.Z., Takahashi M., Ishida Y., Hata J. (2018). Single-cell bioluminescence imaging of deep tissue in freely moving animals. Science.

[84] Zhou Y., Yin K., Dong H., Yang S., Li J., Luo J., Li Y., Yang R. (2021). Long-lasting bioluminescence imaging of the fibroblast activation protein by an amphiphilic block copolymer-based probe. Anal. Chem..

[85] Jiang T., Yang X., Li G., Zhao X., Sun T., Müller R., Wang H., Li M., Zhang Y. (2021). Bacteria-based live vehicle for in vivo bioluminescence imaging. Anal. Chem..

[86] Yang Y., Shao Q., Deng R., Wang C., Teng X., Cheng K., Cheng Z., Huang L., Liu Z., Liu X. (2012). In vitro and in vivo uncaging and bioluminescence imaging by using photocaged upconversion nanoparticles. Angew. Chem. Int. Ed..

[87] Heffern M.C., Park H.M., Au-Yeung H.Y., Van de Bittner G.C., Ackerman C.M., Stahl A., Chang C.J. (2016). In vivo bioluminescence imaging reveals copper deficiency in a murine model of nonalcoholic fatty liver disease. Proc. Natl. Acad. Sci. USA.

[88] Conway M., Xu T., Kirkpatrick A., Ripp S., Sayler G., Close D. (2020). Real-time tracking of stem cell viability, proliferation, and differentiation with autonomous bioluminescence imaging. BMC Biol..

[89] Tögel F., Yang Y., Zhang P., Hu Z., Westenfelder C. (2008). Bioluminescence imaging to monitor the in vivo distribution of administered mesenchymal stem cells in acute kidney injury. Am. J. Physiol. Renal Physiol..

[90] Jiang T., Bai X., Li M. (2024). Advances in the development of bacterial bioluminescence imaging. Annu. Rev. Anal. Chem..

[91] Niu J., Shen L., Huang B., Ye F., Zhao L., Wang H., Deng Y., Tan W. (2020). Non-invasive bioluminescence imaging of HCoV-OC43 infection and therapy in the central nervous system of live mice. Antiviral Res..

[92] Mehle A. (2015). Fiat Luc: Bioluminescence imaging reveals in vivo viral replication dynamics. PLoS Pathog..

[93] Gupta D., Liang X., Pavlova S., Wiklander O.P.B., Corso G., Zhao Y., Saher O., Bost J., Zickler A.M., Piffko A. (2020). Quantification of extracellular vesicles in vitro and in vivo using sensitie bioluminescence imaging. J. Extracell. Vesicles.

[94] Gangadaran P., Li X.J., Lee H.W., Oh J.M., Kalimuthu S., Rajendran R.L., Son S.H., Baek S.H., Singh T.D., Zhu L. (2017). A new bioluminescent reporter system to study the biodistribution of systematically injected tumor-derived bioluminescent extracellular vesicles in mice. Oncotarget.

[95] Ozawa T., Yoshimura H., Kim S.B. (2013). Advances in fluorescence and bioluminescence imaging. Anal. Chem..

[96] Karimi M.A., Lee E., Bachmann M.H., Salicioni A.M., Behrens E.M., Kambayashi T., Baldwin C.L. (2014). Measuring cytotoxicity by bioluminescence imaging outperforms the standard chromium-51 release assy. PLoS ONE.

[97] Arts R., Hartog I., Zijlema S.E., Thijssen V., van der Beelen S.H.E., Merkx M. (2016). Detection of antibodies in blood plasma using bioluminescent sensor proteins and a smartphone. Anal. Chem..

[98] Gräwe A., Merkx M. (2022). Bioluminescence goes dark: Boosting the performance of bioluminescent sensor proteins using complementation inhibitors. ACS Sens..

[99] Tomimuro K., Tenda K., Ni Y., Hiruta Y., Merkx M., Citterio D. (2020). Thread-based bioluminescent sensor for detecting multiple antibodies in a single drop of whole bold. ACS Sens..

[100] Chen L., Bao Y., Denstedt J., Zhang J. (2016). Nanostructured bioluminescent sensor for rapidly detecting thrombin. Biosens. Bioelectron..

[101] Wang D.-D., Jin Q., Zou L.-W., Hou J., Lv X., Lei W., Cheng H.-L., Ge G.-B., Yang L. (2016). A bioluminescent sensor for highly selective and sensitive detection of human carboxylesterase 1 in complex biological samples. Chem. Commun..

[102] Ni Y., Arts R., Merkx M. (2019). Ratiometric bioluminescent sensor proteins based on intramolecular split luciferase complementation. ACS Sens..

[103] Schihada H., Vandenabeele S., Zabel U., Frank M., Lohse M.J., Maiellaro I. (2018). A universal bioluminescence resonance energy transfer sensor design enables high-sensitivity screening of GPCR activation dynamics. Commun. Biol..

[104] Du J., Xu Q., Lu X., Zhang C. (2014). A label-free bioluminescent sensor for real-time monitoring polynucleotide kinase activity. Anal. Chem..

[105] den Hamer A., Dierickx P., Arts R., de Vries J.S.P.M., Brunsveld L., Merkx M. (2017). Bright bioluminescent BRET sensor proteins for measuring intracellular caspase activity. ACS Sens..

[106] Griss R., Schena A., Reymond L., Patiny L., Werner D., Tinberg C.E., Baker D., Johnsson K. (2014). Bioluminescent sensor proteins for point-of-care therapeutic drug monitoring. Nat. Chem. Biol..

[107] Xiong M., Wu Y., Kong G., Lewis W., Yang Z., Zhang H., Xu L., Liu Y., Liu Q., Zhao X. (2023). A semisynthetic bioluminescence sensor for ratiometric imaging of metal ions in vivo using DNAzymes conjugated to an engineered nano-luciferase. Angew. Chem. Int. Ed..

[108] Michielsen C.M.S., van Aalen E.A., Merkx M. (2022). Ratiometric bioluminescent zinc sensor proteins to quantify serum and intracellular free Zn^2+^. ACS Chem. Biol..

[109] Chen F., Warnock R.L., van der Meer J.R., Wegner S.V. (2020). Bioluminescence-triggered photoswitchable bacterial adhesions enable higher sensitivity and dual-readout bacterial biosensors for mercury. ACS Sens..

[110] Viviani V.R., Pelentir G.F., Bevilaqua V.R. (2022). Bioluminescence color-tuning firefly luciferases: Engineering and prospects for real-time intracellular pH imaging and heavy metal biosensing. Biosensors.

[111] Aird E.J., Tompkins K.J., Ramirez M.P., Gordon W.R. (2020). Enhanced molecular tension sensor based on bioluminescence resonance energy transfer (BRET). ACS Sens..

[112] Dash B.S., Das S., Chen J.-P. (2021). Photosensitizer-functionalized nanocomposites for light-activated cancer theranostics. Int. J. Mol. Sci..

[113] Yu H.-H., Deng Q.-P., Zheng Q.-H., Wang Y., Shen J., Zhou J.-H. (2022). Hypericin nanoparticles for self-illuminated photodynamic cytotoxicity based on bioluminescence resonance energy transfer. Int. J. Pharm..

[114] Yang Y., Hou W., Liu S., Sun K., Li M., Wu C. (2018). Biodegradable polymer nanoparticles for photodynamic therapy by bioluminescence resonance energy transfer. Biomacromol..

[115] Yan H., Forward S., Kim K.-H., Wu Y., Hui J., Kashiparekh A., Yun S.-H. (2023). All-natural-molecule, bioluminescent photodynamic therapy results in complete tumor regression and prevents metastasis. Biomaterials.

[116] Al-Ani A.W., Zhang L., Ferreira L., Turyanska L., Bradshaw T.D., Thomas N.R. (2019). Listeria innocua Dps as a nanoplatform for bioluminescence based photodynamic therapy utilizing Gaussia princeps luciferase and zinc protoporphyrin IX. Nanomed. Nanotechnol. Biol. Med..

[117] Lin W., Gong J., Fang L., Jiang K. (2019). A photodynamic system based on endogenous bioluminescence for in vitro anticancer studies. Z. Anorg. Allg. Chem..

[118] Kim E.H., Park S., Kim Y.K., Moon M., Park J., Lee K.J., Lee S., Kim Y.-P. (2020). Self-luminescent photodynamic therapy using breast cancer targeted proteins. Sci. Adv..

[119] Shramova E.I., Filimonova V.P., Frolova A.Y., Pichkur E.B., Fedotov V.R., Konevega A.L., Deyev S.M., Proshkina G.M. (2023). HER-2-specific liposomes loaded with proteinaceous BRET pair as a promising tool for targeted self-excited photodynamic therapy. Eur. J. Pharm. Biopharm..

[120] Hsu C.-Y., Chen C.-W., Yu H.-P., Lin Y.-F., Lai P.-S. (2013). Bioluminescence resonance energy transfer using luciferase-immobilized quantum dots for self-illuminated photodynamic therapy. Biomaterials.

[121] Kim Y.R., Kim S., Choi J.W., Choi S.Y., Lee S.-H., Kim H., Hahn S.K., Koh G.Y., Yun S.H. (2015). Bioluminescence-activated deep-tissue photodynamic therapy of cancer. Theranostics.

[122] Yang K., Wang C., Liu C., Ding S., Tian F., Li F. (2019). Bioluminescence-initiated photodynamic therapy bridged on high-luminescent carbon dots-conjugated protoporphyrin IX. J. Mater. Sci..

[123] Fan D., Wang T., Hu J., Zhou L., Zhou J., Wei S. (2021). Plasmid DNA-based bioluminescence-activated system for photodynamic therapy in cancer treatment. ChemMedChem.

[124] Ng J., Henriquez N., MacRobert A., Kitchen N., Williams N., Bown S. (2022). Bioluminescence-activated photodynamic therapy for luciferase transfected, grade 4 astrocytoma cells in vitro. Photodiagn. Photodyn. Ther..

[125] Ng J., Henriquez N., Kitchen N., Williams N., Novelli M., Oukrif D., MacRobert A., Bown S. (2024). Suppression of tumour growth from transplanted astrocytoma cells transfected with luciferase in mice by bioluminescence mediated, systemic, photodynamic therapy. Photodiagn. Photodyn. Ther..

[126] Shramova E.I., Chumakov S.P., Shipunova V.O., Ryabova A.V., Telegin G.B., Kabashin A.V., Deyev S.M., Proshkina G.M. (2022). Genetically encoded BRET-activated photodynamic therapy for the treatment of deep-seated tumors. Light Sci. Appl..

[127] Yang Z., Zhu Y., Dong Z., Hao Y., Wang C., Li Q., Wu Y., Feng L., Liu Z. (2022). Engineering bioluminescent bacteria to boost photodynamic therapy and systemic anti-tumor immunity for synergistic cancer treatment. Biomaterials.

[128] Packer A.M., Roska B., Häusser M. (2013). Targeting neurons and photons for optogenetics. Nat. Neurosci..

[129] Arrenberg A.B., Stainier D.Y.R., Baier H., Huisken J. (2010). Optogenetic control of cardiac function. Science.

[130] Berglund K., Birkner E., Augustine G.J., Hochgeschwender U. (2013). Light-emitting channelrhodopsins for combined optogenetic and chemical-genetic control of neurons. PLoS ONE.

[131] Berglund K., Clissold K., Li H.E., Wen L., Park S.Y., Gleixner J., Klein M.E., Lu D., Barter J.W., Rossi M.A. (2016). Luminopsins integrate opto-and chemogenetics by using physical and biological light sources for opsin activation. Proc. Natl. Acad. Sci. USA.

[132] Tung J.K., Gutekunst C.-A., Gross R.E. (2015). Inhibitory luminopsins: Genetically-encoded bioluminescent opsins for versatile, scalable, and hardware-independent optogenetic inhibition. Sci. Rep..

[133] Gomez-Ramirez M., More A.I., Friedman N.G., Hochgeschwender U., Moore C.I. (2020). The bioluminescent-optogenetic in vivo response to coelenterazine is proportional, sensitive, and specific in neocortex. J. Neurosci. Res..

[134] Crepso E.L., Bjorefeldt A., Prakash M., Hochgeschwender U. (2021). Bioluminescent optogenetics 2.0: Harnessing bioluminescence to activate photosensory proteins in vitro and in vivo. J. Vis. Exp..

[135] Björefeldt A., Murphy J., Crespo E.L., Lambert G.G., Prakash M., Ikefuama E.C., Friedman N., Brown T.M., Lipscombe D., Moore C.I. (2024). Efficient opto- and chemogenetic control in a single molecule driven by FRET-modified bioluminescence. Neurophotonics.

[136] Slaviero A.N., Gorantla N., Simkins J., Crespo E.L., Ikefuama E.C., Tree M.O., Prakash M., Björefeldt A., Barnett L.M., Lambert G.G. (2024). Engineering luminopsins with improved coupling efficiencies. Neurophotonics.

[137] Petersen E.D., Sharkey E.D., Pal A., Shafau L.O., Zenchak-Petersen J., Peña A.J., Aggarwal A., Prakash M., Hochgeschwender U. (2022). Restoring function after severe spinal cord injury through bioluminescent-optogenetics. Front. Neurol..

[138] English A.W., Berglund K., Carrasco D., Goebel K., Gross R.E., Isaacson R., Mistretta O.C., Wynans C. (2021). Bioluminescent optogenetics: A novel experimental therapy to promote axon regeneration after peripheral nerve injury. Int. J. Mol. Sci..

[139] Ecanow A., Berglund K., Carrasco D., Isaacson R., English A.W. (2022). Enhancing motor and sensory axon regeneration after peripheral nerve injury using bioluminescent optogenetics. Int. J. Mol. Sci..

[140] Zenchak J.R., Palmateer B., Dorka N., Brown T.M., Wagner L.-M., Medendorp W.E., Petersen E.D., Prakash M., Hochgeschwender U. (2020). Bioluminescence-driven optogenetic activation of transplanted neural precursor cells improves motor deficits in a Parkinson’s disease mouse model. J. Neuro. Res..

[141] Ding J., Lu J., Zhang Q., Xu Y., Xu Y., Song B., Wu Y., Shi H., Chu B., Wang H. (2023). Camouflage nanoparticles enable in situ bioluminescence-driven optogenetic therapy of retinoblastoma. ACS Nano.

[142] Adir O., Albalak M.R., Abel R., Weiss L.E., Chen G., Gruber A., Staufer O., Kurman Y., Kaminer I., Shklover J. (2022). Synthetic cells with self-activating optogenetic proteins communicate with natural cells. Nat. Commun..

[143] He L., Huang Z., Huang K., Chen R., Nguyen N.T., Wang R., Cai X., Huang Z., Siwko S., Walker J.R. (2021). Optogenetic control of non-apoptotic cell death. Adv. Sci..

[144] Calabretta M.M., Gregucci D., Martínez-Pérez-Cejuela H., Michelini E. (2022). A luciferase mutant with improved brightness and stability for whole-cell bioluminescent biosensors and in vitro biosensing. Biosensors.

[145] Yeh A.H.-W., Norn C., Kipnis Y., Tischer D., Pellock S.J., Evans D., Ma P., Lee G.R., Zhang J.Z., Anishchenko I. (2023). De novo design of luciferases using deep learning. Nature.

[146] Xie W.J., Liu D., Wang X., Zhang A., Wei Q., Nandi A., Dong S., Warshel A. (2023). Enhancing luciferase activity and stability through generative modeling of natural enzyme sequences. Proc. Natl. Acad. Sci. USA.

[147] Avci P., Gupta A., Sadasivam M., Vecchio D., Pam Z., Pam N., Hamblin M.R. (2013). Low-level laser (light) therapy (LLLT) in skin: Stimulating, healing, restoring. Semin. Cutan. Med. Surg..

